# Processing of Strawberry Juice Using High-Power Ultra-Sound (HPU) and Pulsed Electric Field (PEF): Synergistic Effects on Quality, Color and Anthocyanins Revealed by Chemometrics

**DOI:** 10.3390/foods15193519

**Published:** 2026-10-01

**Authors:** Anica Bebek Markovinović, Višnja Stulić, Predrag Putnik, Tibor Janči, Zoran Herceg, Amin Mousavi Khaneghah, Branimir Pavlić, Danijela Bursać Kovačević

**Affiliations:** 1 Faculty of Food Technology and Biotechnology, University of Zagreb, Pierottijeva 6, 10000 Zagreb, Croatia; anica.bebek@gmail.com (A.B.M.); visnja.stulic@pbf.unizg.hr (V.S.); tibor.janci@pbf.unizg.hr (T.J.); zoran.herceg@pbf.unizg.hr (Z.H.); danijela.bursac.kovacevic@pbf.unizg.hr (D.B.K.); 2 Department of Food Technology, University North, Trg Dr. Žarka Dolinara 1, 48000 Koprivnica, Croatia; 3 Faculty of Biotechnologies (BioTech), ITMO University, 9 Lomonosova Street, 191002 Saint Petersburg, Russia; mousavi@itmo.ru; 4 Halal Research Center of IRI, Iran Food and Drug Administration, Ministry of Health and Medical Education, Tehran 1415845371, Iran; 5 Faculty of Technology, University of Novi Sad, Bul. Cara Lazara 1, 21000 Novi Sad, Serbia; bpavlic@uns.ac.rs

**Keywords:** color stability, electrical conductivity, hydroxymethylfurfural (HMF), juice processing, non-thermal technology, storage stability

## Abstract

Minimally processed, non-thermal technologies are increasingly used to preserve fruit juice quality and safety while extending shelf life. This study evaluated high-power ultrasound (HPU; 25% amplitude, 50% pulse, 2.5–7.5 min) combined with pulsed electric field (PEF; 30 kV cm^−1^, 100 Hz, 1.5–4.5 min), applied in the HPU → PEF sequence, as a hurdle strategy for strawberry juice stored for 7 days at 4 °C. The aim was to assess effects on anthocyanin content, color, and physicochemical properties (conductivity, browning index, dissolved oxygen, HMF) and to identify optimal processing conditions using a chemometric approach. Storage significantly affected all parameters (*p* ≤ 0.01), increasing conductivity, browning index, and total color difference while reducing anthocyanin, oxygen, and HMF levels. Among HPU–PEF combinations, the mildest treatment (HPU 2.5 min + PEF) preserved the highest anthocyanin content (19.48 ± 0.05 mg 100 mL^−1^), lowest conductivity, browning index, and HMF, while more intensive treatments caused progressive degradation. Optimization identified HPU 2.5 min + PEF 1.5 min (no storage) as maximizing anthocyanin retention (20.07 mg 100 mL^−1^), while minimal HMF (4.29 mg L^−1^) required HPU 3.5 min + PEF 1.5 min. Short-duration HPU–PEF combinations thus provide an effective mild-intensity hurdle strategy for maintaining fresh-like strawberry juice quality.

## 1. Introduction

Today, consumers increasingly prefer minimally processed, additive-free foods that are safe, retain their biological and functional properties and offer extended shelf life [1]. Pasteurization remains the standard and most widely applied method for ensuring the microbiological safety and shelf stability of fruit juices, including strawberry juice, and it typically involves a single thermal step without the addition of industrial additives or extensive reformulation of the product. Nonetheless, pasteurization presents certain technological drawbacks: heat treatment can compromise nutritional, sensory, and phytochemical qualities, leading to browning, hydroxymethylfurfural (HMF) formation, color changes, and loss of bioactive compounds [2]. During storage, non-enzymatic reactions, such as Maillard reactions and acid-catalyzed sugar degradation, are primarily responsible for juice browning, since heat inactivates enzymes that normally contribute to quality deterioration [3].

Anthocyanins are water-soluble flavonoid pigments responsible for the characteristic red color of strawberries and their derived products. Pelargonidin-3-glucoside is the predominant anthocyanin in strawberry fruit and juice, typically accounting for the large majority of total anthocyanin content, with smaller contributions from cyanidin-3-glucoside and other minor derivatives [4]. Strawberry juice generally contains total monomeric anthocyanin concentrations in the range of approximately 15–35 mg 100 mL^−1^, depending on cultivar, ripeness stage, and processing conditions [5]. Beyond their contribution to color, and thus to consumer acceptance, anthocyanins are of considerable nutritional interest due to their strong antioxidant capacity and associations with anti-inflammatory, cardioprotective, and potential anticarcinogenic effects reported in dietary and in vitro studies [6]. However, anthocyanins are highly labile compounds, prone to degradation via hydrolysis, oxidation, and condensation reactions, and their stability is strongly influenced by pH, temperature, light exposure, dissolved oxygen, and the presence of enzymes such as polyphenol oxidase and peroxidase [7]. Because conventional thermal pasteurization accelerates several of these degradation pathways, preserving anthocyanin content and color stability during processing and storage represents a key technological challenge, and a central quality criterion, in the development of minimally processed, non-thermally treated strawberry juice [8].

Beyond the quality losses associated with thermal processing itself, fruit juices, including strawberry juice, remain highly susceptible to microbial and enzymatic spoilage throughout production, distribution, and storage, particularly when subjected to inadequate pasteurization, temperature abuse, or extended shelf life under refrigeration [9]. Spoilage in juices typically manifests as microbial growth (yeasts, molds, and lactic or acetic acid bacteria), fermentation off-flavors, gas production, turbidity, and enzymatic browning driven by residual polyphenol oxidase and peroxidase activity, alongside progressive degradation of pigments and vitamins that reduces both sensory acceptability and nutritional value [10]. These phenomena represent a significant source of food loss and economic waste across the juice supply chain, particularly for minimally processed and ‘fresh-like’ products that forgo intensive thermal or chemical preservation in order to retain nutritional and sensory quality [11].

To meet consumer demands, the food industry increasingly applies hurdle technology, which combines multiple processing methods in a strategic sequence to enhance both safety and quality [12]. By synergistically combining techniques, each process can be applied under milder conditions than when used alone, yielding microbiologically stable products with preserved functionality [13]. For instance, previous research suggests that high-power ultrasound (HPU) and pulsed electric field (PEF) together could synergistically improve anthocyanin stability, reduce browning, inactivate microorganisms and enzymes, and preserve key physicochemical properties of strawberry juice [14,15].

Non-thermal technologies, including PEF, HPU, high hydrostatic pressure (HHP), and cold plasma (CP), have been extensively studied as alternatives to thermal pasteurization in juice processing [16]. Each technology presents distinct advantages and constraints: HHP effectively inactivates microorganisms and enzymes with minimal impact on nutritional quality, but requires costly, batch-limited equipment that constrains industrial scalability [17]. CP offers a low-temperature, chemical-free option for surface decontamination, but its effects on liquid matrices such as juices, particularly on pigment stability, remain inconsistent and less established [18]. In contrast, HPU and PEF are both continuous-flow-compatible, comparatively low-cost technologies with well-documented efficacy in microbial inactivation and bioactive compound extraction from fruit matrices [19,20]. HPU generates cavitation bubbles that collapse, producing strong shear forces that disrupt residual plant cell structures within the juice, facilitating the release of cell-wall- and vacuole-bound anthocyanins into the soluble fraction and thereby increasing their measurable concentration [19]. Similarly, PEF uses short, high-voltage pulses to induce electroporation, disrupting intact cell membranes present in the juice matrix and promoting the release of intracellular compounds, including pigments and enzymes [20]. This release process is directly relevant to preservation outcomes: increased liberation of bound anthocyanins can enhance their apparent content immediately after treatment, but exposure of these labile pigments to the surrounding medium (e.g., dissolved oxygen, enzymes, reactive species) may also accelerate their subsequent degradation during storage. Understanding this dual role of cell disruption, as both a potential enhancer of pigment availability and a factor influencing degradation kinetics, is therefore central to evaluating HPU and PEF as preservation strategies for strawberry juice. Combining the two technologies as a hurdle strategy is expected to achieve microbial and enzymatic inactivation while enhancing anthocyanin extractability at milder individual intensities than either technology applied alone [14,15].

Despite this rationale, the combined application of HPU and PEF in strawberry juice, a delicate, anthocyanin-rich matrix, remains underexplored. Our previous study established the effects of the PEF → HPU sequence on anthocyanin content, color parameters, and physicochemical properties of strawberry juice under a comparable experimental design [21]. Considered in isolation, however, that study could establish only the outcomes of one specific treatment order, not whether reversing the sequence would alter the degree of synergism between HPU and PEF, since cavitation-induced structural changes may differently affect subsequent electroporation efficiency depending on whether HPU precedes or follows PEF. No prior study, including our own, has systematically evaluated how the sequence of application affects pigment stability and juice quality through a direct, matched comparison.

The present study addresses this gap by applying the same technologies, treatment durations, and response variables in the reverse (HPU → PEF) order, allowing us to determine whether processing order influences anthocyanin retention, color stability, and physicochemical quality, and, if so, in which direction and to what extent. Because the optimal processing conditions cannot be assumed to be identical between sequences, a separate optimization procedure was necessary to identify the HPU and PEF durations that minimize quality deterioration specifically within the HPU → PEF sequence, enabling comparison of these optimized outcomes against those previously established for the PEF → HPU sequence.

## 2. Materials and Methods

### 2.1. Production of Strawberry Juices

Strawberries, particularly the ’Albion’ cultivar (*Fragaria x ananassa* Duch.), were obtained from Jagodar-HB d.o.o. (Donja Lomnica, Zagreb County). Upon arrival at the laboratory, the fruits were cleaned, destemmed, dried and stored at −18 °C until use. Prior to juicing, they were thawed overnight in a refrigerator. Strawberry juice was then produced using a Kuvings B6000 (VerVita d.o.o., Zagreb, Croatia) cold press with a power of 240 W, a rotation speed of 60 rpm, and a filter diameter of 0.2.

### 2.2. High-Power Ultrasound (Hpu) and Pulsed Electric Field (Pef) Treatment of Strawberry Juices

Following a predefined experimental design (Table 1) and considering optimized process parameters from previous studies, strawberry juice samples were processed using a combination of HPU and PEF technologies.

In addition to the 18 HPU–PEF treatment combinations, untreated strawberry juice samples (control) were included in the experimental design. Control samples consisted of freshly pressed strawberry juice that underwent no HPU or PEF treatment, but were otherwise processed and handled identically to the treated samples, including storage in sterile, tightly sealed glass bottles in the dark at 4 °C. As with the treated samples, control samples were analyzed both immediately after juicing (0 days) and after 7 days of refrigerated storage, with two independent replicates per storage time point, to allow direct comparison between untreated and HPU–PEF-treated juice under equivalent storage conditions.

The HPU and PEF treatment parameters used in this study (HPU: 25% amplitude, 50% pulse, 2.5–7.5 min; PEF: 30 kV cm^−1^, 100 Hz, 1.5–4.5 min) were selected based on optimization results from our previous studies on strawberry juice [22,23]. The same parameter ranges were used in our previous PEF → HPU study [21] to enable a direct comparison of treatment sequences. Keeping the processing parameters and durations identical was a deliberate methodological choice to ensure that any differences in juice quality could be attributed specifically to the treatment order rather than to differences in processing intensity.

A storage temperature of 4 °C was applied, consistent with standard refrigerated storage conditions for non-thermally treated, minimally processed fruit juices, which, unlike thermally pasteurized products, rely on continuous cold-chain maintenance rather than heat-based microbial inactivation to preserve safety and quality [24]. A 7-day storage period was selected to reflect the short-refrigerated shelf-life window typically associated with raw or minimally processed juices lacking thermal or chemical preservation, and to allow direct comparison with our previous study evaluating the reverse PEF → HPU sequence under identical storage conditions [21].

**Figure 1:**
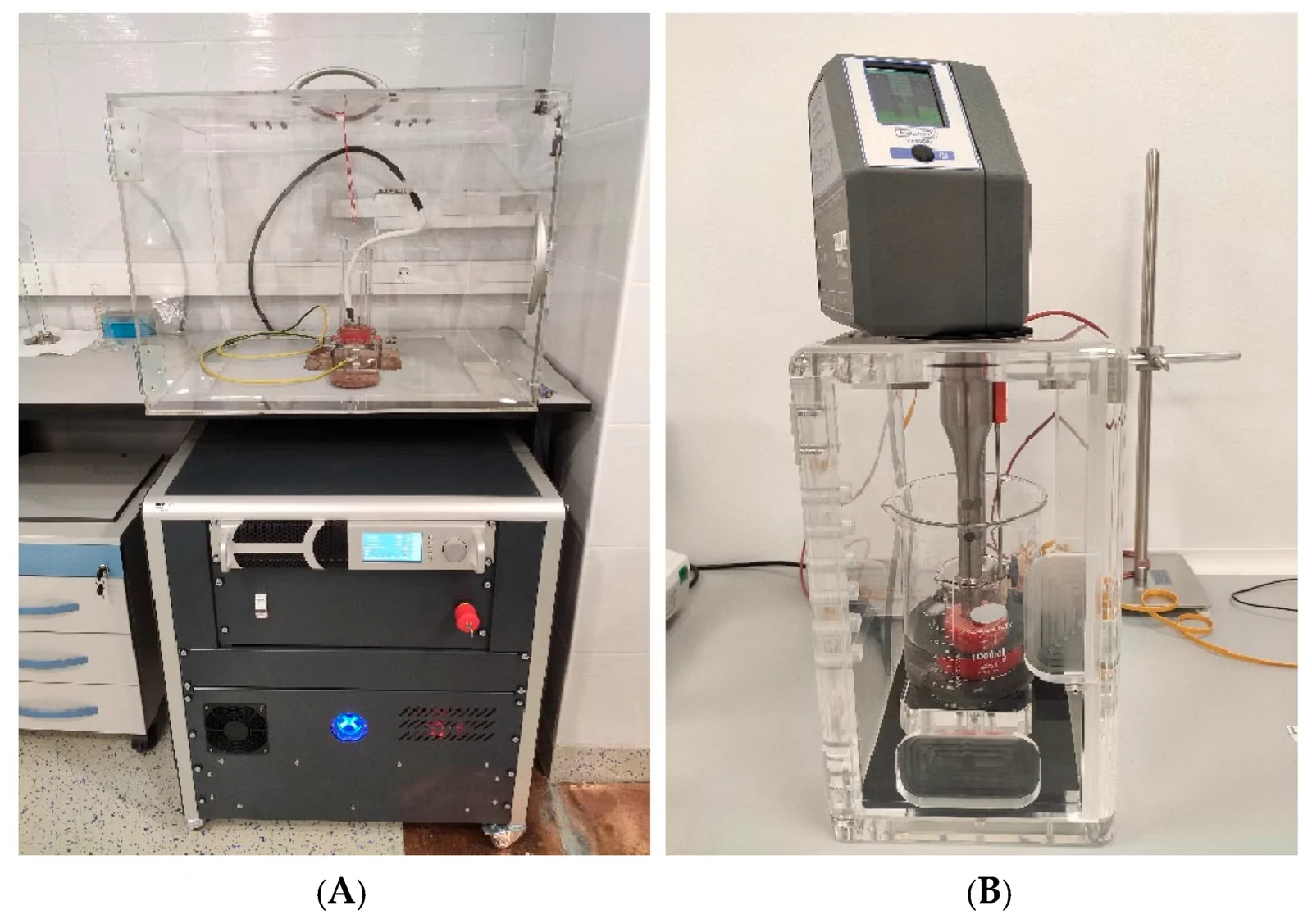
Pulsed electric field device HVG60/1 PEF (Impel d.o.o., Zagreb, Croatia) (**A**) and high-power ultrasonic processor Hielscher UP400St (Hielscher Ultrasonics GmbH, Teltow, Germany) (**B**).

The Hielscher UP400St high-power sonicator (400 W, 24 Hz), equipped with a DN22 titanium sonotrode (546 mm^2^) (Figure 1), was used according to the previously described method [22]. Strawberry juice samples were treated at an amplitude of 25% and a pulse of 50% for duration of 2.5, 5 and 7.5 min. Each 200 mL sample was processed in a glass beaker immersed in an ice-cold water bath to minimize temperature increase during HPU treatment. The sonicator was equipped with a digital thermometer to monitor the sample temperature throughout the process.

Following HPU treatment, the strawberry juices were subjected to PEF treatment using the HVG60/1 PEF device (Impel d.o.o., Zagreb, Croatia) (Figure 1). This device consisted of a 200 mL treatment chamber with two parallel stainless-steel electrodes (68 mm in diameter) spaced 25 mm apart. PEF treatment was applied for 1.5, 3 and 4.5 min at an electric field strength of 30 kV cm^−1^, a pulse frequency of 100 Hz, and a pulse width of 1 µs. During PEF treatment, the temperature was monitored using an infrared thermometer (PCE-777, PCE-Instruments, Manchester, UK).

The temperature of the treated juices was monitored both before and after HPU and PEF treatments. Prior to HPU treatment, the average sample temperature was 15.66 °C, increasing slightly to 19.00 °C after treatment. Similarly, before PEF treatment, the average temperature was 17.40 °C and increased to 17.90 °C after treatment. As the temperature remained relatively stable throughout, its influence on the observed variables was not considered in this study. Following treatments, one batch of juices was analyzed immediately, while a second batch was stored at 4 °C for 7 days prior to analysis. All samples were stored in sterile, tightly sealed glass bottles in the dark.

### 2.3. Determination of Electrical Conductivity

Electrical conductivity of the juice samples was measured in duplicate before and after treatment using a HI-2030 edge conductivity meter (Hanna Instruments, Woonsocket, RI, USA). Results were expressed in µS/cm.

### 2.4. Determination of Dissolved Oxygen

Dissolved oxygen was measured in duplicate using a SevenGo Duo Pro SG68-FK2 device (Mettler-Toledo GmbH, Greifensee, Switzerland). Results were expressed in mg/L.

### 2.5. Instrumental Color Measurements

Color measurements were performed using a Konica Minolta spectrophotometer (CM-700d, Konica Minolta, Tokyo, Japan) equipped with a D65 illuminant and a 10° standard observer, an 8 mm aperture target mask, a plate, and an open cone. A 20 mL sample was placed in a CR-A504 optical glass cuvette (Konica Minolta, Japan), positioned on the target mask plate, and covered with a CM-A182 zero calibration box (Konica Minolta, Japan) to eliminate ambient light interference.

Color parameters (L*, a*, b*) were recorded, and total color difference (ΔE_ab_), chroma (C*), and hue angle (H*) were calculated as follows:

(1) $$\Delta E_{\mathrm{ab}}=\sqrt{{\Delta L}^{*2}+{\Delta a}^{*2}+\Delta b^{*2}}$$

(2) $$C*=\sqrt{a^{*2}+b^{*2}}$$

(3) $$H*=\tan^{-1}\left(\frac{b^{*}}{a^{*}}\right)$$

where all ΔL*^2^, Δa*^2^ and Δb*^2^ represent differences relative to the untreated samples. All measurements were performed in triplicate.

### 2.6. Determination of Browning Index (BI) and Hydroxymethylfurfural (HMF) Content

The browning index (BI) was determined spectrophotometrically [25]. Briefly, 5 g of strawberry juice was mixed with 5 mL of 96% ethanol and centrifuged at 6500 rpm for 10 min. The supernatant was separated; one portion was used for BI determination and the other for HMF analysis.

For the BI determination, absorbance was measured at 420 nm, using distilled water as a blank. BI was calculated as:

(4) $$BI=A_{420\mathrm{nm}}\cdot DF$$

where A_420_ is the absorbance at 420 nm and DF is the dilution factor.

HMF content was determined spectrophotometrically based on the thiobarbituric acid reaction [25]. Briefly, 2 mL of supernatant (diluted if necessary), 2 mL of 12% trichloroacetic acid, and 2 mL of 0.025 M thiobarbituric acid were added to a glass tube. The mixture was vortexed and incubated at 40 °C for 50 min. After cooling under running tap water, absorbance was measured at 443 nm. A blank was prepared using distilled water instead of the supernatant. HMF content was quantified using a calibration curve (up to 20 mg/L) and expressed as mg HMF per 100 mL of sample.

### 2.7. Determination of Monomeric Anthocyanins

The Monomeric anthocyanins were determined from extracts prepared by weighing 5 g of juice into a 50 mL Erlenmeyer flask and adding 20 mL of extraction solvent (1% formic acid in 80% methanol, *v*/*v*). The mixture was subjected to ultrasonic extraction in a Sonorex Digitec ultrasonic bath (Bandelin electronic GmbH, Berlin, Germany) at 50 °C for 15 min. After extraction, samples were filtered (Whatman No. 5 filter paper, pore size 2.5 μm; Whatman plc/Cytiva, Maidstone, UK) into 25 mL volumetric flasks, brought to volume with extraction solvent, and stored at 4 °C until analysis [26].

Monomeric anthocyanins were determined using the pH differential method [27]. Briefly, 1 mL of extract was mixed with 4 mL of pH 1.0 buffer (0.025 M potassium chloride), and another 1 mL with 4 mL of pH 4.5 buffer (0.4 M sodium acetate). After 20 min, absorbance was measured at 520 and 700 nm using an LLG-uniSPEC 2 spectrophotometer (Lab Logistics Group GmbH, Meckenheim, Germany), with deionized water as a blank.

Monomeric anthocyanin content was calculated using the following equation:

(5) $$\frac{A\times MW\times DF\times 10^{3}}{\varepsilon \times l}$$

where A = (A_520_ − A_700_)_pH 1.0_ − (A_520_ − A_700_)_pH 4.5_; MW (molecular weight) = 433.2 g mol ^−1^ for pelargonidin-3-glucoside; DF = dilution factor; l = path length of the cuvette (1 cm); ε = molar absorptivity of pelargonidin-3-glucoside (15,600 L cm^−1^ mol^−1^); and 1000 is the conversion factor from g to mg. Results were expressed as pelargonidin-3-glucoside equivalents (Pg-3-G; mg 100 mL^−1^).

### 2.8. Statistical Analysis

The experiment followed a full-factorial, randomized design with two independent experimental replicates per treatment combination (i.e., each of the 18 unique HPU × PEF × storage combinations was independently prepared and processed in duplicate), yielding a total of n = 36 independent experimental units, as shown in Table 1. These experimental replicates are distinct from the analytical replicate measurements described in Section 2.3, Section 2.4, Section 2.5, Section 2.6 and Section 2.7 (e.g., duplicate or triplicate instrumental readings per experimental unit), which assess measurement precision rather than process variability.

The dependent variables included: (i) monomeric anthocyanins (ANT; mg 100 mL^−1^); (ii) conductivity (COND; µS cm^−1^); (iii) browning index (BI); (iv) hydroxymethylfurfural (HMF; mg L^−1^); (v) CIELab color variables (L, a, b, C, H, ΔE_ab_), and (vi) dissolved oxygen (O_2_; mg L^−1^). Independent variables were: (i) HPU treatment time (2.5, 5.0 and 7.5 min); (ii) PEF treatment time (1.5, 3.0 and 4.5 min); and (iii) storage duration (0 and 7 days). Descriptive statistics summarized the experimental data set. Treatment effects were analyzed using multivariate analysis of variance (MANOVA). Pearson’s correlation coefficients were calculated to assess relationships between variables. Hierarchical Ward cluster analysis evaluated similarities among samples. The Kruskal–Wallis test was applied for nonparametric analysis. Statistical significance was set at α ≤ 0.05. Linear regression was used to develop predictive models, retaining only statistically significant predictors (*p* ≤ 0.05).

All analyses were performed using IBM SPSS Statistics (v.24), and the experimental design was generated using Statgraphics Centurion^®^ (StatPoint Technologies, Inc., Warrenton, VA, USA).

To identify processing conditions that optimize juice quality, multiple linear regression models were first fitted for each response variable (anthocyanin content, conductivity, browning index, HMF, and ΔE_ab_) as a function of HPU time, PEF time, and their interaction, retaining only statistically significant terms (*p* ≤ 0.05). These predictive models were then used within a desirability-function-based optimization procedure (Statgraphics Centurion^®^) to identify the combination of HPU and PEF treatment times, within the experimentally tested range but not necessarily corresponding to the discrete factor levels used in the factorial design, that maximized anthocyanin retention or minimized conductivity, browning index, HMF, and color change, respectively. Optimization was carried out using response surface methodology (RSM). The adequacy of the fitted models was checked using the adjusted coefficient of determination (R^2^adj), the Durbin–Watson statistic and lack-of-fit tests. A non-significant lack-of-fit was taken as an indication that the models described the experimental data satisfactorily, while Durbin–Watson values within the acceptable range suggested that residual autocorrelation was not a concern. Variance inflation factors (VIF) were also examined to check for possible multicollinearity and model overparameterization.

## 3. Results and Discussion

### 3.1. Utilization of Chemometrics for Assessing Processing Effects Compared to Control Samples

**Figure 2:**
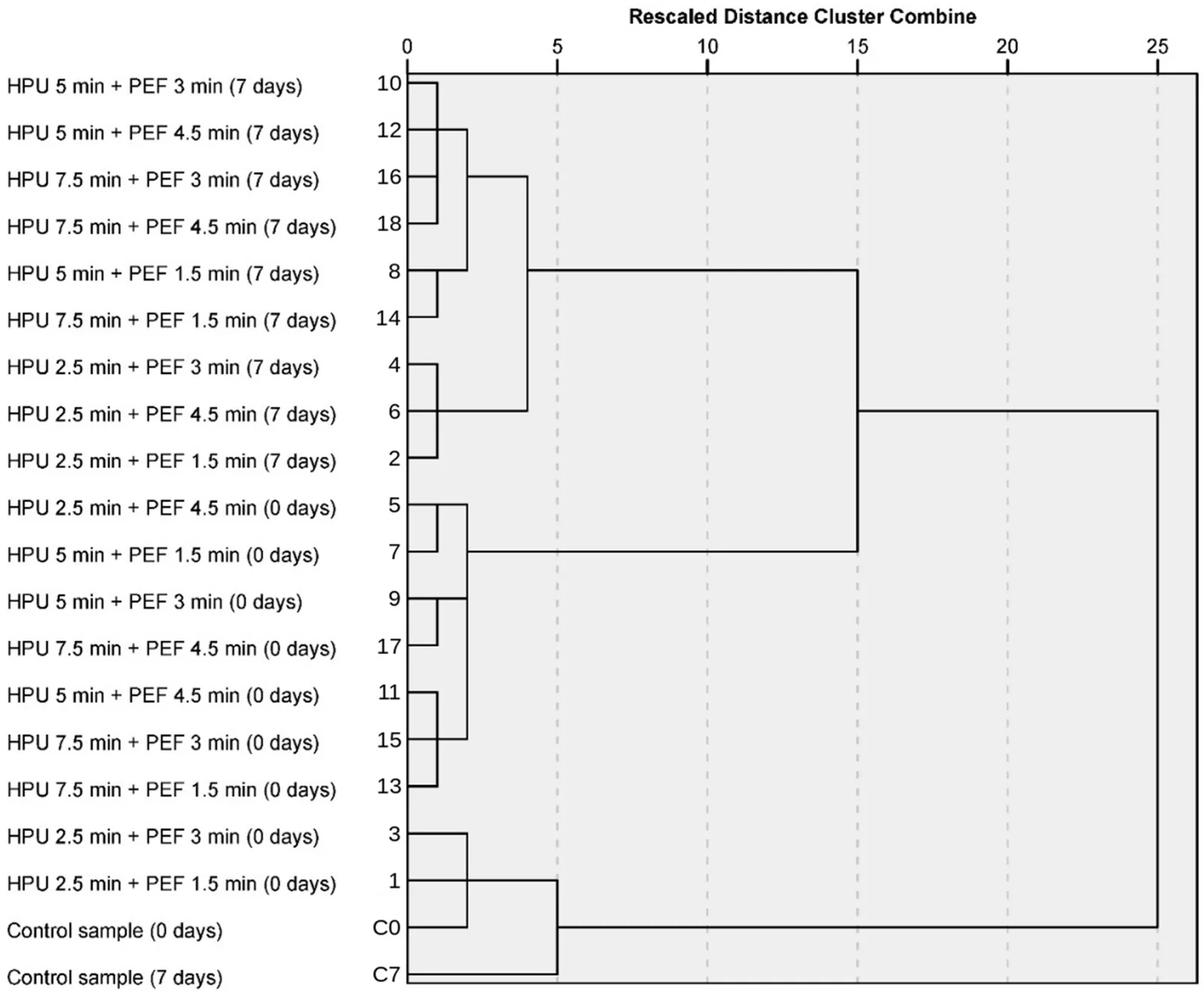
Hierarchical cluster analysis of averaged and standardized samples.

To comprehensively evaluate similarities among samples and the combined effects of processing and storage, a standardized dataset comprising treatment parameters (type and duration of HPU–PEF processing and storage time) and quality attributes (monomeric anthocyanins, browning index, hydroxymethylfurfural, electrical conductivity, dissolved oxygen, and CIELab color parameters L, a, b, C, H, ΔE_ab_) was subjected to Ward’s hierarchical cluster analysis (Figure 2).

It should be emphasized that the 0-day, freshly pressed control represents the target quality reference throughout this study, consistent with the goal of producing a juice that remains ‘fresh-like’ after processing and storage. The 7-day stored control, in contrast, is not used as a quality standard, but rather as a baseline reflecting the natural, unmitigated quality decline of untreated juice under refrigeration alone; its purpose is to isolate the specific contribution of HPU–PEF processing to quality retention, by allowing comparison of how closely treated, stored juice approaches the 0-day fresh reference relative to how far the untreated, stored control itself deviates from that same reference. Accordingly, the finding that 7-day control samples clustered relatively close to the 0-day reference (Section 3.1) indicates that refrigerated storage alone causes only limited degradation over this period, which in turn sets a relatively demanding benchmark: for HPU–PEF processing to provide a clear practical advantage, treated samples must remain as close to, or closer to, the fresh 0-day reference than the untreated stored control does, rather than merely avoiding degradation relative to an already-degraded standard.

It is important to note that proximity to the control samples in the cluster analysis reflects the overall magnitude of change across the combined set of measured variables and does not by itself indicate whether that change is desirable or undesirable; some parameters improve with processing (e.g., anthocyanin retention relative to more intensive treatments), while others deteriorate (e.g., increased conductivity, browning, or color change). Within the cluster of freshly analyzed samples (0 days), those treated with milder processing conditions (HPU 2.5 min combined with PEF 1.5–3.0 min) grouped most closely with the control samples. In this instance, closer proximity to control is a favorable outcome, as it indicates that these mild treatments best preserved the juice’s native anthocyanin content, color, and physicochemical profile relative to the fresh, untreated product, consistent with the goal of a ‘fresh-like’ juice. In contrast, samples subjected to more intensive treatments (longer HPU and/or PEF durations) formed distinct subclusters farther from the control, reflecting greater cumulative deviation from the fresh juice profile; here, greater distance from control is an unfavorable outcome, driven primarily by increased conductivity, browning index, and color change (ΔE_ab_), as confirmed by the parameter-specific results in Table 2. These findings align with the known sensitivity of anthocyanins, which are highly unstable and prone to degradation under external stressors; therefore, shorter and less intensive treatments are more favorable for their retention [28]. Such effects can also accelerate oxidative and non-enzymatic reactions, leading to changes in color parameters, browning index, and HMF formation [29,30].

Interestingly, the control samples stored for 7 days clustered closely with freshly analyzed control samples and mildly treated samples, indicating relatively good stability of untreated juice under the applied storage conditions. From an industrial perspective, this suggests that short-term storage (up to 7 days at 4 °C) does not drastically compromise juice quality, and that mild HPU–PEF treatments can maintain properties comparable to fresh juice. This is a favorable outcome, as it implies that minimal processing can achieve quality retention without introducing significant deviations from the original product profile.

Similarly, the finding that 7-day stored control samples clustered closely with fresh control and mildly treated samples indicates a favorable outcome specifically with respect to overall quality retention during short-term storage, rather than an absence of any measurable change: individual parameters such as anthocyanin content and dissolved oxygen still declined significantly during this period (Table 3), even though the sample’s overall multivariate profile remained relatively close to the fresh, unprocessed reference. This supports the hypothesis that HPU and PEF treatments primarily influence the kinetics of quality changes during storage, rather than causing large immediate differences. The divergence among stored samples likely reflects variations in enzyme inactivation efficiency, oxygen incorporation, and structural modifications of the juice matrix, all of which contribute to the progression of degradation reactions [31,32].

The distinct separation of treated samples from controls, regardless of storage time, further confirms that HPU–PEF processing induces measurable changes in juice properties, even when mild conditions are applied. Although these changes were subtle at the 7-day endpoint evaluated here, each is mechanistically linked to processes that would be expected to continue progressing over longer storage periods, with implications for the juice’s extended stability.

Regarding anthocyanins, processing-induced cell disruption releases pigments from the vacuole into the juice matrix, increasing their exposure to dissolved oxygen, residual enzymes, and reactive species; while this exposure did not yet produce statistically significant losses beyond storage-driven degradation within 7 days, the same exposure pathway is expected to continue driving anthocyanin degradation over an extended shelf life, meaning that treatments causing greater initial cell disruption may exhibit accelerated anthocyanin loss upon longer storage, even if differences are not yet apparent at 7 days.

Regarding electrical conductivity, the observed increase reflects the release of ions and soluble intracellular constituents into the juice matrix. Because this release process, once initiated by cavitation and electroporation, does not reverse during storage, conductivity is expected to plateau rather than decline further; however, elevated conductivity indicates a matrix with greater ionic mobility, which can promote or accelerate other degradative electrochemical and enzymatic reactions (e.g., oxidation) during subsequent storage, indirectly affecting long-term pigment and color stability even though conductivity itself is not a direct spoilage indicator.

Regarding color parameters, particularly ΔE_ab_, the progressive increase observed here reflects the combined effects of anthocyanin degradation, browning reactions, and pigment-matrix interactions. Since browning reactions (Maillard-type and enzymatic) are cumulative and typically continue, rather than plateau, over extended non-thermal storage, the modest ΔE_ab_ increases observed at 7 days likely represent an early stage of a progressive color deterioration process; treatments producing higher ΔE_ab_ or browning index values at this early time point would therefore be expected to reach perceptible or unacceptable color change thresholds (ΔE_ab_ > 6) sooner upon extended storage than milder treatments.

Taken together, while none of the measured changes compromised juice quality within the 7-day window evaluated in this study, the mechanistic links between cell disruption, ionic release, and oxidative exposure indicate that milder HPU–PEF treatments are likely to be more advantageous for maintaining juice quality should the shelf life be extended beyond the period tested here. Confirming this would, however, require future studies incorporating longer storage durations.

**Figure 3:**
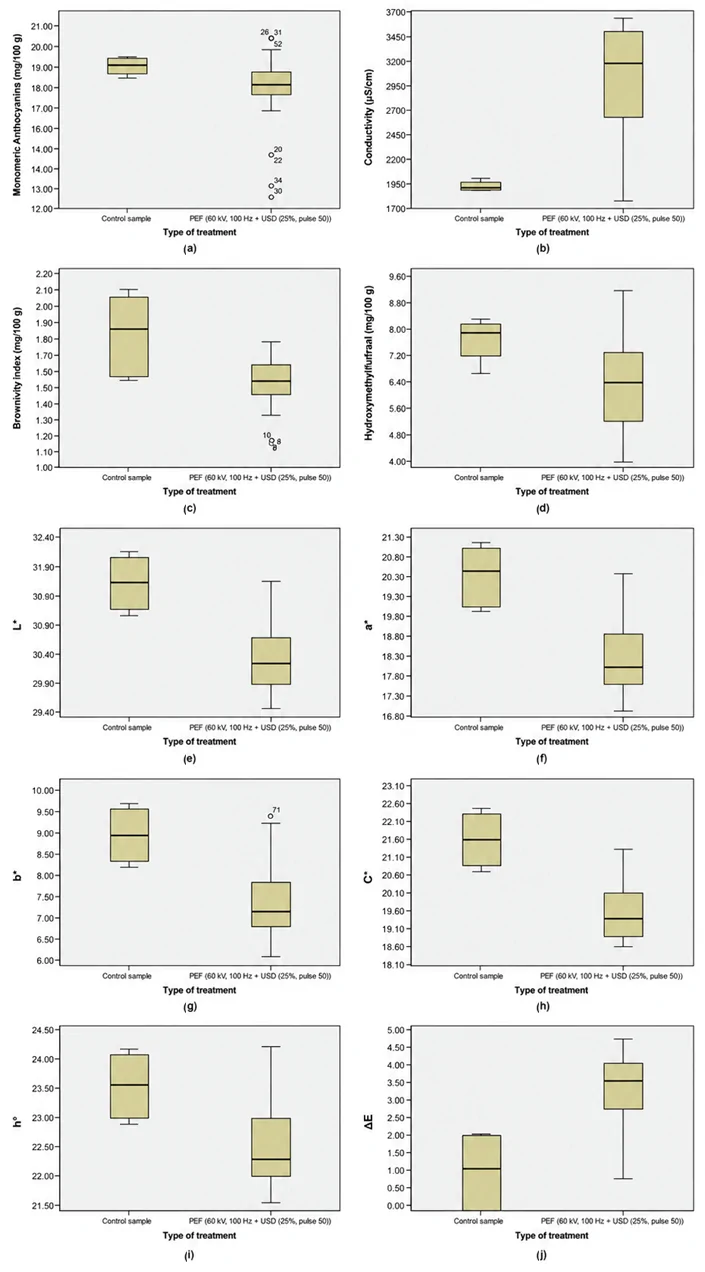
Box plots of the quality parameters of the control and PEF (60 kV, 100 Hz) + USD (25%, pulse 50)-treated samples: (**a**) monomeric anthocyanins (mg 100 g-1); (**b**) electrical conductivity (µS cm-1); (**c**) browning index; (**d**) hydroxymethylfurfural (mg 100 g-1); (**e**) L*, lightness; (**f**) a*, redness (+)/greenness (−); (**g**) b*, yellowness (+)/blueness (−); (**h**) C*, chroma (colour saturation); (**i**) h°, hue angle; (**j**) ΔE, total colour difference relative to the control. Boxes show the interquartile range with the median line, whiskers show the range excluding outliers, and circles (○) mark outliers, labelled by case number.

Table 2 presents the significance values from the Kruskal–Wallis test comparing the combined HPU + PEF treated samples with the control samples. The results show that the treated juices differed from the controls mainly in electrical conductivity and selected CIELab color parameters (L*, a*, b*, C*, and ΔE_ab_). The median values of these variables are illustrated in Figure 3.

Compared to the controls, the treated samples had higher conductivity values, while the control samples consistently showed lower median conductivity. This increase in conductivity can be attributed to the effects of non-thermal processing technologies. Specifically, ultrasound-induced cavitation and PEF-induced electroporation disrupt cellular structures, promoting the release of intracellular components into the extracellular medium and increasing ionic mobility and overall conductivity [16].

These findings are consistent with previous reports [33], which observed significantly higher conductivity in treated grapefruit and orange juices compared to untreated samples.

Regarding color characteristics, the control samples generally showed higher median values for most CIELab parameters (except H* and L*), while ΔE_ab_ values were lower in controls than in treated samples (Figure 3). This suggests that the combined HPU + PEF treatment induced measurable, statistically significant changes in certain color attributes of the juice. However, these changes did not correspond to anthocyanin degradation, increased browning, or HMF formation, indicating that the applied treatments preserved key quality parameters despite modifying color perception.

No statistically significant differences were observed between treated and control samples for several other measured variables, further supporting the conclusion that HPU + PEF processing induces selective rather than extensive modifications in juice properties. Overall, the results demonstrate that while combined non-thermal treatments influence conductivity and specific color parameters, they do so without compromising the general chemical quality and stability of the juice.

### 3.2. The Influence of High-Power Ultrasound (HPU) Combined with Pulsed Electric Field (PEF) on the Physicochemical Properties of Strawberry Juices During Storage

The presence of oxygen in juices can cause detrimental changes in the content of bioactive compounds, particularly anthocyanins. These compounds are highly unstable and sensitive to various factors, including pH, temperature, light, and oxygen exposure [34]. Additionally, oxygen can further accelerate color deterioration in strawberry juices [35].

Table 3 summarizes the effects of storage and non-thermal processing technologies (HPU and PEF), applied individually and in combination, on conductivity, dissolved oxygen, browning index and hydroxymethylfurfural content in strawberry juice. Storage had a significant effect (*p* ≤ 0.01) on all measured parameters. Conductivity increased after 7 days of storage, which can be attributed to the progressive release of intracellular constituents into the extracellular matrix. This phenomenon is likely a consequence of structural damage to cell membranes induced by non-thermal treatments, particularly electroporation and cavitation, which facilitate mass transfer processes. The observed increase suggests that storage acts as a continuation phase of extraction, where previously induced cellular disruption enables sustained diffusion of ions and soluble compounds [36].

When considering the individual effects of processing technologies, both HPU and PEF significantly influenced conductivity (*p* ≤ 0.01), although with distinct patterns. In HPU-treated samples, conductivity increased significantly from 2.5 to 5 min, after which no further increase was observed, indicating a plateau effect. A similar trend was noted for PEF, where conductivity increased up to 3 min, with no statistically significant differences thereafter. These findings are consistent with previous reports indicating that prolonged ultrasound treatment enhances conductivity due to improved extraction efficiency, but only up to a threshold beyond which equilibrium is reached. This plateau can be explained by the exhaustion of extractable intracellular material, as previously described [37,38]. In combined HPU + PEF treatments, conductivity changes depended strongly on treatment intensity. While most combinations resulted in increased conductivity, the most intensive treatment (7.5 min HPU + extended PEF duration) did not produce significant additional increases, confirming that maximal extraction had already been achieved. Notably, shorter combined treatments (e.g., 2.5 min HPU + 3.0 min PEF) were sufficient to reach conductivity values comparable to more intensive regimes, highlighting the efficiency of milder processing conditions. Furthermore, comparison with previously published results [21] indicates that the HPU → PEF sequence achieves higher conductivity under milder conditions than the reverse sequence (PEF → HPU), suggesting a synergistic enhancement when cavitation precedes electroporation.

**Table 3:** Influence of storage, HPU and PEF treatments on the conductivity, dissolved oxygen, browning index, and HMF content in strawberry juices.

Variables	n	COND	O_2_	BI	HMF
**Storage**		***p*** **≤ 0.01 ^†^**	***p*** **≤ 0.01 ^†^**	***p*** **≤ 0.01 ^†^**	***p*** **≤ 0.01 ^†^**
0 days	18	2948.3 ± 28.3 ^b^	7.74 ± 0.07 ^a^	1.55 ± 0.01 ^b^	5.32 ± 0.05 ^a^
7 days	18	3305.6 ± 28.3 ^a^	4.58 ± 0.07 ^b^	1.64 ± 0.01 ^a^	4.21 ± 0.05 ^b^
Average	36	3127.0 ± 28.3	6.16 ± 0.07	1.59 ± 0.01	4.77 ± 0.05
**HPU**		***p*** **≤ 0.01 ^†^**	***p*** **≤ 0.01 ^†^**	***p*** **= 0.07 ^‡^**	***p*** **≤ 0.01 ^†^**
2.5 min	12	2913.2 ± 34.7 ^b^	6.67 ± 0.09 ^a^	1.56 ± 0.02 ^a^	4.43 ± 0.06 ^c^
5.0 min	12	3270.8 ± 34.7 ^a^	5.80 ± 0.09 ^b^	1.62 ± 0.02 ^a^	4.82 ± 0.06 ^b^
7.5 min	12	3196.8 ± 34.7 ^a^	6.02 ± 0.09 ^b^	1.60 ± 0.02 ^a^	5.05 ± 0.06 ^a^
Average	36	3126.9 ± 34.7	6.16 ± 0.09	1.59 ± 0.02	4.77 ± 0.06
**PEF**		***p*** **≤ 0.01 ^†^**	***p*** **= 0.09 ^‡^**	***p*** **= 0.14 ^‡^**	***p*** **= 0.67 ^‡^**
1.5 min	12	2936.5 ± 34.7 ^b^	6.17 ± 0.09 ^a^	1.58 ± 0.02 ^a^	4.79 ± 0.06 ^a^
3.0 min	12	3235.0 ± 34.7 ^a^	6.30 ± 0.09 ^a^	1.58 ± 0.02 ^a^	4.73 ± 0.06 ^a^
4.5 min	12	3209.3 ± 34.7 ^a^	6.02 ± 0.09 ^a^	1.62 ± 0.02 ^a^	4.79 ± 0.06 ^a^
Average	36	3126.9 ± 34.7	6.16 ± 0.09	1.59 ± 0.02	4.77 ± 0.06
**HPU + PEF**		***p*** **≤ 0.01 ^†^**	***p*** **= 0.26 ^‡^**	***p*** **= 0.02 ^†^**	***p*** **= 0.67 ^‡^**
2.5 min + 1.5 min	12	2307.0 ± 87.0 ^b^	6.92 ± 0.17 ^a^	1.55 ± 0.03 ^b^	4.49 ± 0.10 ^a^
2.5 min + 3.0 min	12	3190.0 ± 87.0 ^a^	6.61 ± 0.17 ^a^	1.53 ± 0.03 ^b^	4.37 ± 0.10 ^a^
2.5 min + 4.5 min	12	3242.5 ± 87.0 ^a^	6.50 ± 0.17 ^a^	1.61 ± 0.03 ^a^	4.44 ± 0.10 ^a^
Average	36	2913.2 ± 77.0	6.67 ± 0.17	1.56 ± 0.03	4.43 ± 0.10
**HPU + PEF**		***p*** **≤ 0.01 ^†^**	***p*** **= 0.31 ^‡^**	***p*** **= 0.25 ^‡^**	***p*** **≤ 0.01 ^†^**
5.0 min + 1.5 min	12	3200.0 ± 60.0 ^b^	5.99 ± 0.15 ^a^	1.61 ± 0.03 ^a^	4.42 ± 0.10 ^b^
5.0 min + 3.0 min	12	3295.0 ± 60.0 ^a^	5.79 ± 0.15 ^a^	1.67 ± 0.03 ^a^	5.05 ± 0.10 ^a^
5.0 min + 4.5 min	12	3317.5 ± 60.0 ^a^	5.62 ± 0.15 ^a^	1.58 ± 0.03 ^a^	4.98 ± 0.10 ^a^
Average	36	3270.8 ± 60.0	5.80 ± 0.15	1.62 ± 0.03	4.82 ± 0.10
**HPU + PEF**		***p*** **= 0.06 ^‡^**	***p*** **≤ 0.01 ^†^**	***p*** **= 0.06 ^‡^**	***p*** **≤ 0.01 ^†^**
7.5 min + 1.5 min	12	3302.5 ± 55.0 ^a^	5.61 ± 0.12 ^b^	1.59 ± 0.03 ^a^	5.45 ± 0.10 ^a^
7.5 min + 3.0 min	12	3220.0 ± 55.0 ^a^	6.50 ± 0.12 ^a^	1.54 ± 0.03 ^a^	4.76 ± 0.10 ^b^
7.5 min + 4.5 min	12	3068.0 ± 55.0 ^a^	5.93 ± 0.12 ^b^	1.69 ± 0.03 ^a^	4.95 ± 0.10 ^b^
Average	36	3196.8 ± 5.0	6.01 ± 0.12	1.60 ± 0.03	5.05 ± 0.10

The results are expressed as mean ± standard error. Values represented with different letters are statistically different at *p* ≤ 0.05; ^†^ significant factor in multifactor analysis; ^‡^ not significant factor in multifactor analysis. COND-conductivity (µS cm^−1^); O_2_-oxygen content (mg L^−1^); BI-browning index; HMF-hydroxymethylfurfural (mg L^−1^). Average values represent group means calculated across the corresponding factor levels (n = 36) and serve as the reference point for the post hoc statistical comparisons among treatment levels reported within each block.

Dissolved oxygen content was significantly reduced during storage (*p* ≤ 0.01), likely due to oxygen consumption in oxidative reactions and limited oxygen diffusion in sealed systems, consistent with earlier findings [39]. Among processing treatments, HPU tended to reduce oxygen content with increasing treatment time, particularly up to 5 min, which can be attributed to its well-documented degassing effect [40]. In contrast, PEF alone had no significant effect on oxygen levels (*p* = 0.09), indicating its limited role in gas removal.

Combined HPU + PEF treatments generally did not significantly affect dissolved oxygen content, except under the most intensive conditions. This may be explained by two factors: (i) the inability of PEF to contribute to degassing, and (ii) possible re-dissolution of oxygen into the juice between sequential treatments. These findings are consistent with previous observations showing that the order of application (HPU + PEF vs. PEF + HPU) does not significantly influence oxygen content, except at extreme processing intensities [21].

Color is a critical factor influencing consumer preference for strawberry products, with the browning index (BI) serving as a key indicator of undesirable browning. Color stability, expressed as BI, was significantly affected by storage (*p* ≤ 0.01), with higher BI values observed after 7 days, indicating increased browning. Since both oxygen and HMF content decreased during storage, their contribution to browning can be considered negligible in this context. Instead, the increase in BI is more plausibly linked to anthocyanin degradation, as supported by the significant reduction in anthocyanin content reported in Table 4. This aligns with previous studies identifying anthocyanin instability as a key driver of color deterioration in strawberry products [41].

Non-thermal treatments had limited effects on BI, with most individual and combined treatments showing no significant changes. The only notable exception was the combination of 2.5 min HPU with extended PEF duration (up to 4.5 min), where a significant increase in BI was observed. However, despite this increase, the mildest combined treatment exhibited the lowest overall BI values, indicating better color preservation. When correlated with anthocyanin data, an inverse relationship between BI and anthocyanin content is evident, further supporting the role of pigment degradation in browning development. Compared to the HPU + PEF sequence, the PEF + HPU combination reported in previous work appears to be milder, generally resulting in reduced BI values except under the most intensive conditions [21].

HMF content was significantly reduced during storage (*p* ≤ 0.01), suggesting possible degradation or transformation during storage [42]. This contrasts with previous findings for PEF + HPU treatments, where storage increased HMF levels, indicating that processing sequence may influence HMF stability. Regarding individual treatments, HPU led to a significant increase in HMF with increasing treatment duration, likely due to localized temperature rises during cavitation. In contrast, PEF had no significant effect on HMF formation (*p* = 0.67).

Combined HPU + PEF treatments exhibited complex and treatment-dependent trends. For mild conditions (2.5 min HPU), no significant changes in HMF were observed with increasing PEF duration. At intermediate intensity (5 min HPU), increasing PEF duration from 1.5 to 3 min significantly increased HMF, while further extension had no additional effect. Conversely, for the most intensive HPU treatment (7.5 min), increasing PEF duration resulted in a decrease in HMF content, suggesting possible degradation or transformation mechanisms at higher energy inputs. Overall, the lowest HMF levels were observed under the mildest combined treatment conditions, indicating that process optimization can minimize thermal-like degradation effects.

Comparison with the reverse sequence (PEF + HPU) [21] reveals that HPU + PEF is generally more aggressive in terms of HMF formation, but also more efficient, as similar effects can be achieved under milder conditions. This highlights the importance of process sequencing and parameter optimization in balancing extraction efficiency and quality preservation.

### 3.3. The Influence of High-Power Ultrasound (HPU) Combined with Pulsed Electric Field (PEF) on the Anthocyanins and Colour Stability of Strawberry Juices During Storage

Table 4 presents the effects of storage and non-thermal processing technologies (HPU and PEF), applied individually and in combination, on anthocyanin content and color parameters (L*, a*, b*, C*, H*, and ΔE_ab_) of strawberry juice. Storage at 4 °C for 7 days significantly reduced anthocyanin content (*p* ≤ 0.01), confirming the susceptibility of these pigments to degradation even under refrigerated conditions [34].

This result is consistent with previous studies reporting anthocyanin losses in strawberry juices during cold storage [22]. The reduction in anthocyanin content was accompanied by an increase in browning index (Table 3), indicating an inverse relationship between pigment stability and browning development. This supports the hypothesis that anthocyanin degradation plays a central role in the formation of undesirable brown coloration during storage [34].

Processing treatments further affected anthocyanin stability. In general, both individual and combined applications of HPU and PEF decreased anthocyanin content as treatment intensity increased. This trend was especially evident in combined HPU + PEF treatments, where longer secondary PEF treatment resulted in progressive anthocyanin degradation. The only exception was the intermediate treatment (HPU 5 min + PEF 1.5–4.5 min), where no statistically significant changes in anthocyanin content were observed, suggesting a possible balance between enhanced extraction and degradation processes.

Considering mean values, the highest anthocyanin content (19.48 ± 0.05 mg 100 mL^−1^) was observed under the mildest combined treatment (HPU 2.5 min + PEF), indicating that gentle processing conditions favorable for pigment retention. These findings align with literature reports highlighting the detrimental effects of prolonged ultrasound exposure of anthocyanins due to localized energy input, free radical formation, and potential temperature increases [22,43]. In contrast to the PEF → HPU sequence reported previously [21], which often showed a parabolic trend (initial increase followed by degradation), the HPU → PEF sequence applied in this study predominantly resulted in a continuous decline in anthocyanin levels, indicating a more aggressive impact on pigment stability.

Color parameters were strongly affected by both storage and processing conditions (*p* ≤ 0.01). Storage significantly reduced lightness (L*), confirming the visual darkening of juice samples. This change is consistent with the observed decrease in anthocyanins and the simultaneous increase in browning (Table 3). Similar reductions in L* values during storage have been reported in fruit-based systems, including strawberry and orange products [24,44].

For individual treatments, HPU caused a progressive decrease in all color parameters (L*, a*, b*, C*, H*) with increasing treatment time, indicating deterioration of color intensity and hue stability. In contrast, PEF showed a non-linear (parabolic) effect: treatment up to 3 min significantly enhanced color attributes, while further prolongation led to their decline. This suggests that moderate PEF conditions may enhance pigment extractability or dispersion, whereas excessive treatment promotes degradation.

Combined HPU and PEF treatments exhibited treatment-dependent behavior. Under mild conditions (HPU 2.5 min), increasing PEF duration to 3 min significantly improved all color parameters, while further extension to 4.5 min resulted in their decline, mirroring the pattern observed for PEF alone. A similar trend was observed for the HPU 5 min + PEF combination, although changes in b* and H* were not statistically significant. The most intensive treatment (HPU 7.5 min + PEF) showed minimal sensitivity to moderate increases in PEF duration, but further extension led to an increase in color parameter values, suggesting complex interactions between degradation and structural modifications at higher energy inputs.

Overall, these results indicate that color stability is highly dependent on treatment intensity and sequence, with moderate processing conditions providing optimal preservation of visual quality. Although few studies have examined the combined effects of HPU and PEF on strawberry juice color, previous work on PEF-treated fruit juices confirms that processing parameters critically influence CIELab values [45].

Total color difference (ΔE_ab_) was significantly affected by storage (*p* ≤ 0.01), with higher values observed after 7 days, reflecting perceptible color changes. This is consistent with the reduction in L* and anthocyanin content. Similar increases in ΔE_ab_ during storage have been reported for strawberry products [46]. Although treatment duration in combined HPU + PEF processes did not always significantly affect ΔE_ab_, the highest mean value (3.96 ± 0.09) was observed under the most intensive treatment conditions. Importantly, all ΔE_ab_ values remained below 6, indicating that color differences were noticeable but still within an acceptable range for consumers.

In summary, both storage and processing significantly influence anthocyanin stability and color attributes of strawberry juice. Mild processing conditions, particularly shorter HPU and moderate PEF treatments, appear to offer the best balance between preservation of bioactive compounds and maintenance of desirable color characteristics.

**Table 4:** Influence of storage, HPU and PEF treatments on the anthocyanins and color parameters.

Variables	n	ANT	L*	a*	b*	C*	H*	ΔE_ab_
Storage		*p* ≤ 0.01 ^†^	*p* ≤ 0.01 ^†^	*p* ≤ 0.01 ^†^	*p* ≤ 0.01 ^†^	*p* ≤ 0.01 ^†^	*p* ≤ 0.01 ^†^	*p* ≤ 0.01 ^†^
0 days	18	19.61 ± 0.04 ^a^	31.43 ± 0.01 ^a^	18.31 ± 0.03 ^a^	7.87 ± 0.02 ^a^	19.93 ± 0.03 ^a^	23.26 ± 0.02 ^b^	3.16 ± 0.05 ^b^
7 days	18	18.17 ± 0.04 ^b^	31.03 ± 0.01 ^b^	17.92 ± 0.03 ^b^	7.35 ± 0.02 ^b^	19.37 ± 0.03 ^b^	22.29 ± 0.02 ^a^	3.85 ± 0.05 ^a^
Average	36	18.89 ± 0.04	31.23 ± 0.01	18.12 ± 0.03	7.61 ± 0.02	19.65 ± 0.03	22.78 ± 0.02	3.51 ± 0.05
HPU		*p* ≤ 0.01 ^†^	*p* ≤ 0.01 ^†^	*p* ≤ 0.01 ^†^	*p* ≤ 0.01 ^†^	*p* ≤ 0.01 ^†^	*p* ≤ 0.01 ^†^	*p* ≤ 0.01 ^†^
2.5 min	12	19.48 ± 0.05 ^b^	31.43 ± 0.02 ^a^	18.75 ± 0.03 ^a^	8.00 ± 0.02 ^a^	20.39 ± 0.04 ^a^	23.07 ± 0.03 ^a^	2.73 ± 0.06 ^b^
5.0 min	12	18.79 ± 0.05 ^a^	31.12 ± 0.02 ^b^	17.90 ± 0.03 ^b^	7.41 ± 0.02 ^b^	19.37 ± 0.04 ^b^	22.47 ± 0.03 ^c^	3.81 ± 0.06 ^a^
7.5 min	12	18.40 ± 0.05 ^c^	31.13 ± 0.02 ^b^	17.69 ± 0.03 ^c^	7.43 ± 0.02 ^b^	19.19 ± 0.04 ^c^	22.78 ± 0.03 ^b^	3.96 ± 0.06 ^a^
Average	36	18.89 ± 0.05	31.23 ± 0.02	18.11 ± 0.03	7.61 ± 0.02	19.65 ± 0.04	22.77 ± 0.03	3.50 ± 0.06
PEF		*p* ≤ 0.01 ^†^	*p* ≤ 0.01 ^†^	*p* ≤ 0.01 ^†^	*p* ≤ 0.01 ^†^	*p* ≤ 0.01 ^†^	*p* ≤ 0.01 ^†^	*p* ≤ 0.01 ^†^
1.5 min	12	19.28 ± 0.05 ^a^	31.16 ± 0.02 ^b^	18.01 ± 0.03 ^b^	7.56 ± 0.02 ^b^	19.53 ± 0.04 ^b^	22.73 ± 0.03 ^b^	3.64 ± 0.06 ^a^
3.0 min	12	18.91 ± 0.05 ^b^	31.35 ± 0.02 ^a^	18.34 ± 0.03 ^a^	7.73 ± 0.02 ^a^	19.90 ± 0.04 ^a^	22.83 ± 0.03 ^a^	3.23 ± 0.06 ^b^
4.5 min	12	18.48 ± 0.05 ^c^	31.18 ± 0.02 ^b^	17.99 ± 0.03 ^b^	7.55 ± 0.02 ^b^	19.51 ± 0.04 ^b^	22.76 ± 0.03 ^a,b^	3.64 ± 0.06 ^a^
Average	36	18.89 ± 0.05	31.23 ± 0.02	18.11 ± 0.03	7.61 ± 0.02	19.65 ± 0.04	22.77 ± 0.03	3.50 ± 0.06
HPU +PEF		*p* ≤ 0.01 ^†^	*p* ≤ 0.01 ^†^	*p* ≤ 0.01 ^†^	*p* ≤ 0.01 ^†^	*p* ≤ 0.01 ^†^	*p* = 0.02 ^†^	*p* ≤ 0.01 ^†^
2.5 min + 1.5 min	12	19.80 ± 0.05 ^a^	31.44 ± 0.03 ^b^	18.74 ± 0.06 ^b^	7.97 ± 0.04 ^b^	20.36 ± 0.07 ^b^	23.03 ± 0.04 ^b^	2.76 ± 0.08 ^b^
2.5 min + 3.0 min	12	19.63 ± 0.05 ^b^	31.65 ± 0.03 ^a^	19.30 ± 0.06 ^a^	8.33 ± 0.04 ^a^	21.02 ± 0.07 ^a^	23.31 ± 0.04 ^a^	2.06 ± 0.08 ^c^
2.5 min + 4.5 min	12	19.02 ± 0.05 ^c^	31.20 ±0.03 ^c^	18.22 ± 0.06 ^c^	7.69 ± 0.04 ^c^	19.78 ± 0.07 ^c^	22.89 ± 0.04 ^b^	3.39 ± 0.08 ^a^
Average	36	19.48 ± 0.05	31.43 ± 0.03	18.75 ± 0.06	8.00 ± 0.04	20.39 ± 0.07	23.08 ± 0.04	2.74 ± 0.08
HPU + PEF		*p* = 0.18 ^‡^	*p* ≤ 0.01 ^†^	*p* ≤ 0.01 ^†^	*p* = 0.27 ^‡^	*p* = 0.02 ^†^	*p* = 0.10 ^‡^	*p* = 0.10 ^‡^
5.0 min + 1.5 min	12	18.91 ± 0.17 ^a^	30.98 ± 0.03 ^a^	17.68 ± 0.06 ^b^	7.36 ± 0.04 ^a^	19.15 ± 0.07 ^b^	22.54 ± 0.04 ^a^	4.06 ± 0.12 ^a^
5.0 min + 3.0 min	12	18.58 ± 0.17 ^a^	31.26 ± 0.03 ^b^	18.08 ± 0.06 ^a^	7.46 ± 0.04 ^a^	19.56 ± 0.07 ^a^	22.40 ± 0.04 ^a^	3.60 ± 0.12 ^a^
5.0 min + 4.5 min	12	18.87 ± 0.17 ^a^	31.13 ± 0.03 ^c^	17.93 ± 0.06 ^a^	7.42 ± 0.04 ^a^	19.41 ± 0.07 ^a^	22.48 ± 0.04 ^a^	3.77 ± 0.12 ^a^
Average	36	18.79 ± 0.17	31.12 ± 0.03	17.90 ± 0.06	7.41 ± 0.04	19.37 ± 0.07	22.47 ± 0.04	3.81 ± 0.12
HPU + PEF		*p* ≤ 0.01 ^†^	*p* ≤ 0.01 ^†^	*p* = 0.02 ^†^	*p* = 0.02 ^†^	*p* = 0.02 ^†^	*p* = 0.03 ^†^	*p* ≤ 0.01 ^†^
7.5 min + 1.5 min	12	19.13 ± 0.07 ^a^	31.06 ± 0.03 ^b^	17.60 ± 0.04 ^b^	7.34 ± 0.03 ^b^	19.07 ± 0.05 ^b^	22.63 ± 0.06 ^b^	4.10 ± 0.09 ^a^
7.5 min + 3.0 min	12	18.51 ± 0.07 ^b^	31.13 ± 0.03 ^a,b^	17.64 ± 0.04 ^b^	7.41 ± 0.03 ^b^	19.13 ± 0.05 ^b^	22.79 ± 0.06 ^a,b^	4.02 ± 0.09 ^a^
7.5 min + 4.5 min	12	17.54 ± 0.07 ^c^	31.21 ± 0.03 ^a^	17.83 ± 0.04 ^a^	7.54 ± 0.03 ^a^	19.36 ± 0.05 ^a^	22.92 ± 0.06 ^a^	3.77 ± 0.09 ^a^
Average	36	18.39 ± 0.07	31.13 ± 0.03	17.69 ± 0.04	7.43 ± 0.03	19.19 ± 0.05	22.78 ± 0.06	3.96 ± 0.09

The results are expressed as mean ± standard error. Values represented with different letters are statistically different at *p* ≤ 0.05; ^†^ significant factor in multifactor analysis; ^‡^ not significant factor in multifactor analysis. ANT-monomeric anthocyanins (mg 100 mL^−1^); L*—lightness; a*, b*—CIELab coordinates; C*—chroma; H*—hue; ΔE_ab_—color change.

### 3.4. Optimization of High-Power Ultrasound (Hpu) and Pulsed Electric Field (Pef) Processing Parameters

Optimization of hurdle technology parameters is essential to balance maximal bioactive compound retention with minimal deterioration of physicochemical and sensory properties (color). The results in Table 5 clearly show that processing conditions play a decisive role in determining the quality of strawberry juice treated with combined HPU and PEF technologies.

The optimal conditions for maximizing anthocyanin content (20.07 mg 100 mL^−1^) were achieved at relatively mild processing parameters, HPU treatment for 2.5 min followed by PEF treatment for 1.5 min, without storage. These findings confirm that excessive processing intensity is unnecessary for enhancing anthocyanin extractability and, conversely, may promote degradation. This trend aligns with the results in Table 4, where increasing treatment duration generally led to a decline in anthocyanin levels, highlighting the sensitivity of these compounds to mechanical and oxidative stress.

Similarly, the lowest conductivity (2089.1 µS cm^−1^) and minimal browning index (1.58) were also achieved under the same mild conditions (HPU 2.5 min + PEF 1.5 min), suggesting that limited cell disruption is sufficient for desirable extraction while avoiding excessive release of intracellular electrolytes and subsequent quality deterioration. This indicates that moderate electroporation combined with controlled cavitation provides an optimal balance between permeability enhancement and structural preservation of the juice matrix.

In contrast, minimizing HMF formation (4.29 mg L^−1^) required slightly longer HPU treatment (3.5 min) combined with short PEF exposure (1.5 min), suggesting that HMF formation is influenced by different mechanisms than anthocyanin degradation. This may be due to localized temperature increases and enhanced reaction kinetics during prolonged ultrasonic treatment. Furthermore, optimal color preservation (ΔE_ab_ ≈ 0) was achieved at HPU 3.5 min and PEF 2.0 min, indicating that slight adjustments in processing intensity may be necessary depending on the targeted quality parameter.

A key outcome of this study is that all optimal conditions involved relatively short PEF treatment times (1.5–2.0 min), regardless of the response variable. This observation agrees with previously published work [21], where optimization of the PEF + HPU sequence also indicated that shorter PEF exposure is sufficient to achieve maximal anthocyanin yield and minimal physicochemical changes. However, an important distinction arises when comparing the sequence of technology application. In the HPU → PEF sequence investigated here, optimal HPU treatment times (2.5–3.5 min) were significantly shorter than those reported for the reverse sequence (PEF → HPU), where HPU duration extended up to 7.5 min.

This difference suggests a higher process efficiency of the HPU + PEF sequence, likely due to the initial cavitation-induced disruption of cell structures, which facilitates subsequent electroporation and mass transfer. In contrast, when PEF is applied first, longer ultrasonic treatment may be required to achieve comparable extraction efficiency. These findings highlight the importance of process sequencing as a critical factor in designing hurdle technologies.

Supporting this interpretation, previous studies comparing individual technologies have shown that PEF can be more effective than ultrasound in enhancing anthocyanin extraction under certain conditions [47]. However, the present results demonstrate that when properly combined, HPU and PEF exhibit synergistic effects, enabling efficient extraction at significantly reduced processing intensities.

Overall, the optimization results indicate that mild processing conditions—characterized by short HPU and PEF treatment times—are sufficient to maximize anthocyanin retention while minimizing undesirable changes such as browning, HMF formation, and color degradation. This highlights the potential of carefully designed hurdle technology approaches to improve both the nutritional and sensory quality of fruit juices while maintaining process efficiency.

Considering all five optimized response variables together (Table 5), a clear pattern emerges: three of the five individual optima, anthocyanin content (20.07 mg 100 mL^−1^), conductivity (2089.1 µS cm^−1^), and browning index (1.58), converge on the same mild processing condition, HPU 2.5 min + PEF 1.5 min without storage. Minimal HMF formation (4.29 mg L^−1^) required only a slightly longer HPU duration (3.5 min), while optimal color preservation (ΔE_ab_ ≈ 0) required HPU 3.5 min + PEF 2.0 min after 1 day of storage. Given that four of the five individual optima cluster within a narrow processing window, and three of them coincide exactly at the lowest tested levels of both factors, we identify HPU 2.5 min (25% amplitude, 50% pulse) followed by PEF 1.5 min (30 kV cm^−1^, 100 Hz, 1 µs pulse width) as the single, numerically defined optimum of this study. At this condition, the fitted models predict an anthocyanin content of 20.07 mg 100 mL^−1^, a conductivity of 2089.1 µS cm^−1^ and a browning index of 1.58 (Table 5), while the corresponding experimentally measured means (averaged over 0 and 7 days of storage) were 19.80 ± 0.05 mg 100 mL^−1^, 2307.0 ± 87.0 µS cm^−1^ and 1.55 ± 0.03, respectively (Table 3, Table 4 and Table 6).

Selecting this single condition involves only small, quantifiable trade-offs for the two responses whose individual optima lie outside it (Table 6). For HMF, the content measured at HPU 2.5 min + PEF 1.5 min (4.49 ± 0.10 mg L^−1^) exceeded the model-predicted minimum (4.29 mg L^−1^) by only 0.20 mg L^−1^ and did not differ significantly from the other HPU 2.5 min combinations (*p* = 0.67; Table 3). For color, the lowest ΔE_ab_ among treated samples was measured at HPU 2.5 min + PEF 3.0 min (2.06 ± 0.08), compared with 2.76 ± 0.08 at the recommended condition; this difference of 0.70 units is below ΔE_ab_ = 1, generally regarded as the threshold of visually perceptible color difference, whereas extending PEF from 1.5 to 3.0 min increased conductivity by 883 µS cm^−1^ (+38.3%) and decreased anthocyanin content by 0.17 mg 100 mL^−1^. The model-predicted color optimum (HPU 3.5 min + PEF 2.0 min, ΔE_ab_ ≈ 0) refers to 1 day of storage and therefore does not represent the end of the 7-day shelf life considered here. Compared with the most intensive combination tested (HPU 7.5 min + PEF 4.5 min), the recommended condition retained 2.26 mg 100 mL^−1^ (12.9%) more anthocyanins and yielded browning index, HMF and ΔE_ab_ values lower by 0.14, 0.46 mg L^−1^ and 1.01 units, respectively, while requiring a three-fold shorter total treatment time (4.0 vs. 12.0 min).

Notably, this recommended condition corresponds precisely to the mild end of the treatment range at which the HPU → PEF sequence was shown to offer its clearest practical advantage over the previously investigated PEF → HPU sequence [21] (see Discussion). At this specific processing window, HPU → PEF achieves anthocyanin retention, conductivity, and browning index outcomes at least comparable to those previously optimized for PEF → HPU, while requiring markedly shorter treatment durations, consistent with the process-efficiency advantage attributed to cavitation-first processing. Accordingly, we recommend HPU 2.5 min + PEF 1.5 min (with an operating tolerance of up to HPU 3.0 min and PEF 2.0 min for process control) not only as the optimal condition within the present study, but as the specific processing window in which the HPU → PEF sequence is scientifically and practically justified as the preferable hurdle strategy relative to its reverse-order counterpart.

Considering quality retention, processing intensity, treatment duration, and the stability constraints of the product, HPU 2.5 min followed by PEF 1.5 min is therefore recommended as the single treatment combination most suitable for further scale-up. From a scale-up perspective, HPU 2.5 min + PEF 1.5 min is also the most favorable of the tested combinations. It corresponds to the lowest energy input and the shortest total treatment time (4.0 min per batch, compared with 12.0 min for the most intensive combination), which translates directly into higher throughput and lower operating costs for a given equipment capacity. For PEF, the recommended condition corresponds to 9000 pulses (100 Hz × 90 s) with a cumulative pulse-on time of 9 ms at 30 kV cm^−1^; because PEF efficacy depends on field strength and on the number of pulses received by each volume element rather than on treatment time per se, these values, rather than batch time, should be kept constant when the process is transferred to a continuous treatment chamber. For HPU, time-based parameters are equipment- and volume-specific, so industrial transfer should be based on the acoustic energy density delivered to the juice (e.g., determined calorimetrically), using the value corresponding to 2.5 min treatment of 200 mL in the present setup as the target. The temperature increases during treatment remained small (on average from 15.66 to 19.00 °C during HPU and from 17.40 to 17.90 °C during PEF), indicating that, provided comparable energy densities are maintained, the recommended condition can be applied without additional cooling and without thermally induced quality loss.

The recommended condition must also be considered in light of the product’s stability constraints. During 7 days at 4 °C, storage alone reduced anthocyanin content by 7.3% (from 19.61 to 18.17 mg 100 mL^−1^) and dissolved oxygen by 40.8% (from 7.74 to 4.58 mg L^−1^), while conductivity increased by 12.1% (from 2948.3 to 3305.6 µS cm^−1^), the browning index from 1.55 to 1.64, and ΔE_ab_ from 3.16 to 3.85 (Table 3 and Table 4). Because these storage-driven changes are comparable in magnitude to the differences between processing conditions (e.g., 1.44 vs. up to 2.26 mg 100 mL^−1^ for anthocyanins), the practical value of process optimization lies mainly in the initial quality with which the juice enters storage. By starting from the highest anthocyanin content and the lowest conductivity and browning index, the recommended condition provides the largest quality margin over the 7-day refrigerated shelf life, and all ΔE_ab_ values remained below the threshold of 6 associated with unacceptable color change (Section 3.3). Juice processed at HPU 2.5 min + PEF 1.5 min is therefore best positioned as a chilled, ‘fresh-like’ product with a shelf life of up to 7 days under an unbroken cold chain at ≤4 °C.

Two constraints must nevertheless be addressed before industrial implementation. First, microbial inactivation and residual enzyme activity (e.g., polyphenol oxidase and peroxidase) were not determined in this study. So, the recommended condition is therefore optimized for quality retention, and it remains to be verified that it achieves the microbial safety required for commercial juice (e.g., the 5-log pathogen reduction commonly required for juice processing) and sufficient enzyme inactivation for the intended shelf life. Should the mildest condition prove insufficient in this respect, the present results indicate that treatment intensity should preferably be increased by extending PEF within the HPU 2.5 min series rather than by extending HPU: HPU 2.5 min + PEF 3.0 min still retained 19.63 ± 0.05 mg 100 mL^−1^ anthocyanins and gave the lowest ΔE_ab_ (2.06 ± 0.08), albeit at a higher conductivity (3190.0 ± 87.0 µS cm^−1^), whereas longer HPU treatments consistently lowered anthocyanin content (18.79 and 18.40 mg 100 mL^−1^ at 5.0 and 7.5 min) and increased ΔE_ab_ (3.81 and 3.96) (Table 4). Second, the storage period was limited to 7 days, so the stability of juice processed at the recommended condition over longer refrigerated shelf lives remains to be confirmed.

## 4. Conclusions

This study evaluated the effects of different HPU and PEF treatment durations, applied in the HPU → PEF sequence, on the quality parameters of strawberry juice during 7 days of refrigerated storage (4 °C).

Storage significantly affected all measured parameters (*p* ≤ 0.01), decreasing anthocyanin, dissolved oxygen, and HMF content while increasing conductivity, browning index, and ΔE_ab,_ thereby highlighting its dual role in driving both physicochemical changes and bioactive compound degradation.

A comparison between untreated and treated samples further showed that HPU + PEF processing significantly increased conductivity and overall color change (ΔE_ab_), whereas untreated samples retained higher a*, b*, and C* values, reflecting better preservation of color intensity in the absence of processing.

Among the HPU–PEF combinations, the mildest treatment (HPU 2.5 min + PEF 1.5 min) best preserved juice quality, retaining the highest anthocyanin content (19.80 ± 0.05 mg 100 mL^−1^)—11.4% higher than the most intensive combination tested (HPU 7.5 min + PEF 4.5 min; 17.54 ± 0.07 mg 100 mL^−1^), while also yielding the lowest conductivity, browning index, and HMF content. Consistently, model-based optimization identified HPU 2.5 min + PEF 1.5 min (without storage) as the condition maximizing anthocyanin retention (20.07 mg 100 mL^−1^) and minimizing conductivity (2089.1 µS cm^−1^) and browning index (1.58), whereas minimizing HMF formation (4.29 mg L^−1^) required a slightly longer HPU treatment (3.5 min) combined with a short PEF exposure (1.5 min).

Overall, these findings demonstrate that short-duration HPU–PEF combinations applied in the HPU → PEF sequence constitute an effective mild-intensity hurdle strategy for preserving strawberry juice quality, extending its usable refrigerated shelf life while maintaining bioactive compound content, color stability, and low levels of thermal degradation markers consistent with a ‘fresh-like’ product.

## Figures and Tables

**Table 1:** Full-factorial, randomized experimental design with two independent experimental replicates per treatment combination (n = 36 independent experimental units).

Sample ID	HPU Treatment (Min)	PEF Treatment (Min)	Storage (Days)
1	2.5	1.5	0
2	2.5	1.5	7
3	2.5	3	0
4	2.5	3	7
5	2.5	4.5	0
6	2.5	4.5	7
7	5	1.5	0
8	5	1.5	7
9	5	3	0
10	5	3	7
11	5	4.5	0
12	5	4.5	7
13	7.5	1.5	0
14	7.5	1.5	7
15	7.5	3	0
16	7.5	3	7
17	7.5	4.5	0
18	7.5	4.5	7

**Table 2:** Kruskal–Wallis test statistics for combined technology vs. control samples.

	ANT	COND	BI	HMF	L*	a*	b*	C*	H*	ΔE_ab_	O_2_
Chi-Square	0.018	8.331	0.398	1.171	3.502	9.399	8.331	9.398	3.417	8.854	0.203
df	1	1	1	1	1	1	1	1	1	1	1
Sig.	0.89	≤0.01 *	0.53	0.28	0.06	≤0.01 *	≤0.01 *	≤0.01 *	0.07	≤0.01 *	0.65

* The Kruskal–Wallis test is significant at *p* ≤ 0.05. ANT—Monomeric Anthocyanins (mg 100 mL^−1^); COND—Conductivity (µS cm^−1^); BI—Browning index; HMF—Hydroxymethylfurfural (mg L^−1^); L*—lightness; a*, b*—CIELab coordinates; C*—chroma; H*—hue; ΔE_ab_-color change; O_2_—dissolved oxygen in juice (mg L^−1^).

**Table 5:** Optimal processing parameters for the combination of HPU and PEF technologies to achieve maximum monomeric anthocyanin content and minimum changes in color, conductivity, browning index, and HMF content.

	ANT	COND	BI	HMF	ΔE_ab_
Storage (days)	0.0	0.0	0.0	0.0	1.0
HPU treatment (min)	2.5	2.5	2.5	3.5	3.5
PEF treatment (min)	1.5	1.5	1.5	1.5	2.0
Optimum	20.07	2089.1	1.58	4.29	0.0

ANT—monomeric anthocyanins (mg 100 mL^−1^); COND—conductivity (µS cm^−1^); BI—browning index; HMF—hydroxymethylfurfural (mg L^−1^); ΔE_ab_—color change. PEF—pulsed electric field (30 kV cm^−1^, 100 Hz); and HPU—high-power ultrasound (amplitude 25%, pulse 50%).

**Table 6:** Quality parameters of strawberry juice at the recommended HPU–PEF condition compared with the best-color alternative, the most intensive combination tested, and the model-predicted individual optima.

Parameter	HPU 2.5 Min + PEF 1.5 Min (Recommended)	HPU 2.5 Min + PEF 3.0 Min	HPU 7.5 Min + PEF 4.5 Min	Model-Predicted Optimum ^a^
Total treatment time (min)	4.0	5.5	12.0	–
ANT (mg 100 mL^−1^)	19.80 ± 0.05	19.63 ± 0.05	17.54 ± 0.07	20.07
COND (µS cm^−1^)	2307.0 ± 87.0	3190.0 ± 87.0	3068.0 ± 55.0	2089.1
BI	1.55 ± 0.03	1.53 ± 0.03	1.69 ± 0.03	1.58
HMF (mg L^−1^)	4.49 ± 0.10	4.37 ± 0.10	4.95 ± 0.10	4.29
O_2_ (mg L^−1^)	6.92 ± 0.17	6.61 ± 0.17	5.93 ± 0.12	–
ΔE_ab_	2.76 ± 0.08	2.06 ± 0.08	3.77 ± 0.09	0.0

Measured values are means ± standard error across 0 and 7 days of storage (Table 3 and Table 4). ^a^ Model-predicted individual optima (Table 5) correspond to HPU 2.5 min + PEF 1.5 min at 0 days (ANT, COND, BI), HPU 3.5 min + PEF 1.5 min at 0 days (HMF) and HPU 3.5 min + PEF 2.0 min at 1 day of storage (ΔE_ab_); dissolved oxygen was not optimized. ANT—monomeric anthocyanins; COND—conductivity; BI—browning index; HMF—hydroxymethylfurfural; O_2_—dissolved oxygen; ΔE_ab_—total color change.

## Data Availability

The data that support the findings of this study are available from the corresponding author upon reasonable request.
